# The Convention of Medical Colleges

**Published:** 1879-06

**Authors:** 


### Editorial.

### The Convention of American Medical Colleges.

In compliance with a series of resolutions offered by Professor Gross at the last meeting of the American Medical College Association, held at Buffalo in June, 1878, a call was issued by this Association for a convention of delegates from all duly accredited medical colleges in the United States to meet at Atlanta, Georgia, May 2, 1879. Each medical school was requested to send two delegates, one from its board of trustees, the other from its faculty, and to instruct these representatives so as to authorize their action in behalf of some uniform system of medical teaching more in accordance with the spirit of the age and the standard of education in Europe. When the convention assembled, Prof. S. D. Gross, of the Jefferson Medical College, was called to the chair, and Prof. Starling Loveing, of the Starling Medical College, of Columbus, Ohio, was appointed secretary.

The meeting being organized, Prof. N. S. Davis read the preambles and resolutions adopted at the last meeting of the American Medical College Association, and stated that the object of the convention was to have some authoritative expression of opinion from the colleges in regard to uniformity in medical teaching, and to see if the colleges of this country could not be induced to take a step forward. The students should furnish evidence that they have mastered the ordinary English branches, and should be required to attend three annual courses of medical instruction prior to graduation. The sentiment of the meeting was expressed in the following propositions, the first of which passed after discussion; the second passed unanimously.

First, all medical colleges should require attendance upon three regular courses of lectures during three separate years before admitting students to become candidates for the degree of M. D. Second, the medical colleges should require, before admitting to matriculation, a preliminary examination, such examination to embrace at least the elements of the physicial sciences in addition to a fair English education.

On motion these propositions were directed to be transferred to the American Medical College Association, and that body was apprised of the action of this covention, which adjourned *sine die*.

